# Amentoflavone-Enriched *Selaginella rossii* Warb. Suppresses Body Weight and Hyperglycemia by Inhibiting Intestinal Lipid Absorption in Mice Fed a High-Fat Diet

**DOI:** 10.3390/life12040472

**Published:** 2022-03-24

**Authors:** Hwa Lee, Seona Cho, Soo-Yong Kim, Jeongha Ju, Sang Woo Lee, Sangho Choi, Hulin Li, Renzhe Piao, Ho-Yong Park, Tae-Sook Jeong

**Affiliations:** 1 Industrial Bio-Materials Research Center, Korea Research Institute of Bioscience and Biotechnology (KRIBB), Daejeon 34141, Korea; leehua@kribb.re.kr (H.L.); baby5624@naver.com (S.C.); wjdgk0605@naver.com (J.J.); hypark@kribb.re.kr (H.-Y.P.); 2 International Biological Material Center, KRIBB, Daejeon 34141, Korea; soodole@kribb.re.kr (S.-Y.K.); ethnolee@kribb.re.kr (S.W.L.); decoy0@kribb.re.kr (S.C.); 3 Department of Medical Biotechnology, Yeungnam University, Gyeongsan 38541, Korea; 4 Department of Agronomy, Agriculture College of Yanbian University, Yanji 133000, China; lhlsym@ybu.edu.cn (H.L.); rzpiao@ybu.edu.cn (R.P.)

**Keywords:** *Selaginella rossii*, lipid absorption, hyperglycemia, amentoflavone, fatty acid transport

## Abstract

Many *Selaginellaceae* species are used as traditional medicines in Asia. This study is the first to investigate the anti-obesity and anti-diabetic effects of *Selaginella rossii* (SR) in high-fat diet (HFD)–fed C57BL/6J mice. Seven-day oral administration of ethanol extract (100 mg/kg/day) or ethyl acetate (EtOAc) extract (50 mg/kg/day) from SR improved oral fat tolerance by inhibiting intestinal lipid absorption; 10-week long-term administration of the EtOAc extract markedly reduced HFD-induced body weight gain and hyperglycemia by reducing adipocyte hypertrophy, glucose levels, HbA1c, and plasma insulin levels. Treatment with SR extracts reduced the expression of intestinal lipid absorption-related genes, including *Cd36*, fatty acid-binding protein 6, ATP-binding cassette subfamily G member 8, NPC1 like intracellular cholesterol transporter 1, and ATP-binding cassette subfamily A member 1. In addition, the EtOAc extract increased the expression of protein absorption–related solute carrier family genes, including *Slc15a1*, *Slc8a2*, and *Slc6a9*. SR extracts reduced HFD-induced hepatic steatosis by suppressing fatty acid transport to hepatocytes and hepatic lipid accumulation. Furthermore, amentoflavone (AMF), the primary compound in SR extracts, reduced intestinal lipid absorption by inhibiting fatty acid transport in HFD-fed mice. AMF-enriched SR extracts effectively protected against HFD-induced body weight gain and hyperglycemia by inhibiting intestinal lipid absorption.

## 1. Introduction

Obesity is a chronic metabolic disease that is a major risk factor for diabetes, fatty liver, cardiovascular disease, stroke, and cancer [1,2]. Obesity results in the storage of excess energy as triglycerides (TGs) in adipose tissue (AT) and ectopic fat deposition in the liver, muscle, heart, and pancreas. Dietary fat contributes to increased blood TG levels and obesity. Lipid accumulation in ectopic organs plays a critical role in tissue dysfunction. In particular, hepatic lipid accumulation is an important factor of metabolic complications associated with obesity [3]. Intestinal lipid absorption is necessary for health; however, dysregulated lipid absorption can lead to hyperlipidemia and metabolic diseases [4]. Dietary lipids, including triglycerides (TG), phospholipids, and cholesterol esters, are digested and emulsified by digestive lipases and bile acids in the gastrointestinal lumen [5]. Lipids are further emulsified by hydrolysis and micellar preparation to absorb across the intestinal wall. Lipid hydrolysates solubilized in micelles are absorbed by intestinal transporters, including clusters of differentiation 36 (CD36) or fatty acid transport proteins (FATPs) [6]. Enterocytes that absorb dietary lipids can be stored in cytosolic lipid droplets or secreted in chylomicron particles and are subsequently delivered to tissues by the lymphatic and blood circulatory systems [7]. Hydrolyzed lipoproteins are distributed in the peripheral tissues through blood circulation. Chronic over-nutrition can disrupt the ATs capability of energy storage function and induce ectopic lipid accumulation in non-ATs such as liver and skeletal muscles [8]. Ectopic accumulation of lipids in peripheral tissues leads to profound glucose tolerance with damaged insulin sensitivity in various diseases such as type 2 diabetes and fatty liver disease [9]. 

Orlistat is a representative anti-obesity drug approved for long-term use in clinical therapy [10]. Orlistat reduces fat absorption from the intestine by inhibiting pancreatic lipase activity [11]. Previous studies have demonstrated that 7.5% of individuals with obesity cannot take orlistat over the long term due to serious side effects, including gastrointestinal and musculoskeletal symptoms [12]. Worldwide, over-the-counter weight loss supplements or functional foods are more widely used than prescription drugs for weight loss. Herbal formulations for the mitigation of obesity based on traditional knowledge and usage offer an effective strategy [13]. However, there is little evidence to support the efficacy of many products in promoting weight loss [14]. Therefore, it is important to determine effective natural sources of weight loss that have zero or negligible side effects. 

Many *Selaginellaceae* species have been used as traditional medicines in Asia, such as *Selaginella tamariscina*, *S. pulvinata*, and *S. sinensis*. *S. tamariscina,* in particular, has been reported to possess antioxidant, anti-hyperglycemic, and antihyperlipidemic effects in diabetes by enhancing peroxisome proliferator-activated receptor gamma (PPARγ) expression in AT and insulin receptor substrate 1 (IRS-1) expression in liver and muscle [15,16]. Recently, *S. tamariscina* has been used in cosmetics in Korea based on its anti-aging, anti-wrinkle, and anti-atopic effects [17]. *S. rossii* (SR) has been reported to possess anti-proliferative effects on leukemia U937 cells [18]. However, there are no reports of SR activities on weight loss, metabolic lipids, and glucose homeostasis. We hypothesized that SR extract would help reduce obesity and glucose homeostasis via effects on lipid absorption. Hence, our study aimed to investigate the weight loss and anti-hyperglycemic activities of SR and further elucidate the molecular mechanisms underlying the action of the active compound, amentoflavone (AMF), in high-fat diet (HFD)–fed mice.

## 2. Materials and Methods

### 2.1. Preparation of SR Extracts and High-Performance Liquid Chromatography (HPLC) Analysis

The dried aerial parts of *Selagenella rossii* (Barker) Warb (SR) were obtained from Yanbian University (Yanji, China) delivered by the International Biological Material Research Center of the Korea Research Institute of Bioscience and Biotechnology (KRIBB, Daejeon, Korea). The 50 g dried SR powders were extracted using 500 mL 95% EtOH or EtOAc at 20–25 °C for 48 h, respectively. The EtOH (SRE) and EtOAc (SREA) extracts of SR were concentrated in vacuo to yield a brown residue of 6.0 g and 2.6 g, respectively.

The components of SR extracts were analyzed using a high-performance liquid chromatography-diode array detector (HPLC-DAD) system (Shimadzu Corp., Tokyo, Japan). SR extracts were separated on a Brownlee SPP C18 column (4.6 × 50 mm, 2.7 μm; Perkin Elmer, Inc., Waltham, MA, USA). The compositions of the mobile phase are 0.1% acetic acid in water (mobile phase A) and acetonitrile (mobile phase B). The linear gradient elution program was as follows: 5–50% B at 0–15 min, 50–100% B at 15–20 min, 100% B at 20–25 min, 100–5% B at 25–27 min, and 5% B at 27–30 min. The absorbance of the HPLC profile was 267 nm, and the flow rate was 1.8 mL/min. AMF (Biopurify Phytochemicals Ltd., Chengdu, China) was used as external standards. The purity of AMF was 98.0%. 

### 2.2. Animals and Diets

All protocols in this study were approved by the Animal Care and Use Committee of KRIBB (KRIBB-ACE-19169). Male C57BL/6J mice (4-week-old, Nara Biotech, Pyeongtaek-si, Korea) were maintained under a controlled a 12 h light-dark cycle at 22 ± 2 °C and 50 ± 5% humidity. The mice were free to access to gamma-irradiated diets and autoclaved water in a specific pathogen-free facility in the KRIBB. All experiments were conducted after 2 weeks of acclimation. For oral administration, SR extract was dissolved and diluted in sterile purified water containing 10% polyethylene glycol and 0.5% Tween-80 and AMF was dissolved in 0.1% dimethyl sulfoxide and diluted in sterile purified water containing 10% polyethylene glycol and 0.5% Tween-80.

#### 2.2.1. Experiment 1

After acclimation for two weeks, mice were randomly assigned to 3 groups (*n* = 7): HFD, HFD+SRE, and HFD+SREA. The HFD group was fed a 60 kcal% fat diet (D12492, Research Diet, Inc., New Brunswick, NJ, USA); the HFD+SRE group was fed an HFD with 100 mg∙kg^−1^∙day^−1^ SRE, and the HFD+SREA group was fed an HFD with 50 mg∙kg^−1^∙day^−1^ SREA. SRE and SREA were orally gavaged daily for seven days. The dose of SRE and SREA used in this study was based on previously reported studies [19,20]. An oral fat tolerance test (OFTT) and fecal lipid analysis were conducted on mice.

#### 2.2.2. Experiment 2

To assess the effects after long-term administration of SR extracts, mice were randomly assigned to four groups (*n* = 8): normal diet (ND), HFD, HFD+SRE, and HFD+SREA. The ND group was fed a 10 kcal% fat diet (D12450B, Research Diet, Inc.); The HFD group was fed an HFD; the HFD+SRE group was fed an HFD with 100 mg∙kg^−1^∙day^−1^ SRE, and the HFD+SREA group was fed an HFD with 50 mg∙kg^−1^∙day^−1^ SREA. SRE and SREA were orally gavaged daily for 10 weeks. The plasma profiles, oral glucose tolerance test (OGTT) results, and organ histology were analyzed.

#### 2.2.3. Experiment 3

To assess the effects of AMF, mice were randomly assigned to three groups (*n* = 6): ND, HFD, and HFD+AMF. The ND group was fed an ND; the HFD group was fed an HFD, and the HFD+AMF group was fed an HFD with 10 mg∙kg^−1^∙day^−1^ AMF for seven days. The OFTT was conducted after seven days, and tests for mRNA and protein expression were conducted after 10 days.

### 2.3. OFTT and OGTT

Mice from Experiment 1 or Experiment 3 were fasted 16 h before OFTT, and then olive oil (10 μL/g body weight) was orally administered. Blood samples were collected at 0, 2, and 4 h using heparinized capillaries. The blood was separated, and plasma TG concentration was measured using a TG kit (Asan Pharm. Co. Ltd., Seoul, Korea). 

Mice from Experiment 2 were fasted overnight before OGTT, and then OGTT was conducted as previously described after orogastric gavage of 2 g/kg glucose [21]. Glucose levels were measured using an ACCU-CHEK Active Kit (Roche Diabetes Care, Mannheim, Germany).

### 2.4. Plasma Parameters

The plasma concentrations of TG, total cholesterol (TC), plasma aspartate transaminase (AST), and alanine transaminase (ALT) were measured by individual available kits (Asanpharm Co., Hwaseong-si, Korea). Plasma insulin concentration was measured using an Insulin ELISA kit (Alpco Diagnostics, Salem, NH, USA). HbA1c was measured using an HbA1c detection Kit (Osang Healthcare, Anyang, Korea). The HOMA-IR index was calculated using the following formula: HOMA-IR index = [fasting insulin concentration (ng/mL)] × 24.8 × [fasting glucose concentration (mg/dL)] /405 [22].

### 2.5. Fecal Lipid Profiles

Fecal samples were collected from mice for three days and dried. Dried feces (100 mg) were homogenized, and total lipids were isolated using 5% Triton X-100. Repeated heating for solubilizing the lipids. The solution was centrifuged at 13,500× *g* for 5 min and supernatants were used to measure TG and free fatty acid (FFA; Biomax Co. Ltd., Seoul, Korea).

### 2.6. RNA Preparation and Quantitative Real-Time RT-PCR (qRT-PCR)

The freshly isolated intestine and liver were used for RNA extraction. RNA was extracted using an RNeasy Mini Kit (Qiagen GmbH, Hilden, Germany). Total RNA was used to synthesize cDNA using a cDNA synthesis kit (Thermo Fisher Scientific Baltics, Vilnius, Lithuania). qRT-PCR was carried out using a 7500 real-time PCR system (Applied Biosystems, Foster City, CA, USA) using specific primers (Table S1).

### 2.7. Histological Analysis

The histological analysis was performed using ATs and liver after Experiment 2. For histological hematoxylin-eosin (H&E) staining, freshly isolated ATs and livers were fixed, embedded, and sliced into 4-μm sections. For Oil-red O staining, cryostat sections of livers were fixed and stained with Oil-red O (Sigma-Aldrich, St. Louis, MO, USA) solution. All tissues from each group were analyzed (*n* = 8). At least three sections were taken from each sample for staining, and at least three digital images were obtained from each section using a camera-mounted microscope system (Olympus, Tokyo, Japan). To determine the size of the adipocytes, minimum of 10 adipocytes from one view were measured using MetaMorph^®^ Imaging System (Meta Imaging Software, Sunnyvale, CA, USA). 

### 2.8. Western Blotting Analysis 

The protein samples from liver or intestine tissue lysates were performed to Western blotting analysis according to standard procedures using anti-CD36 (Santa Cruz Biotechnology, Santa Cruz, CA, USA) and anti-GAPDH (Bioss Inc., Woburn, MA, USA) antibodies.

### 2.9. Data Analysis

Statistical analysis was performed using JMP^®^ software (SAS Institute Inc., Cary, NC, USA) with a one-way analysis of variance. Values were given as means ± SE. A *p*-value < 0.05 was considered as significant difference.

## 3. Results

### 3.1. HPLC Analysis of SR Extracts 

**Figure 1:**
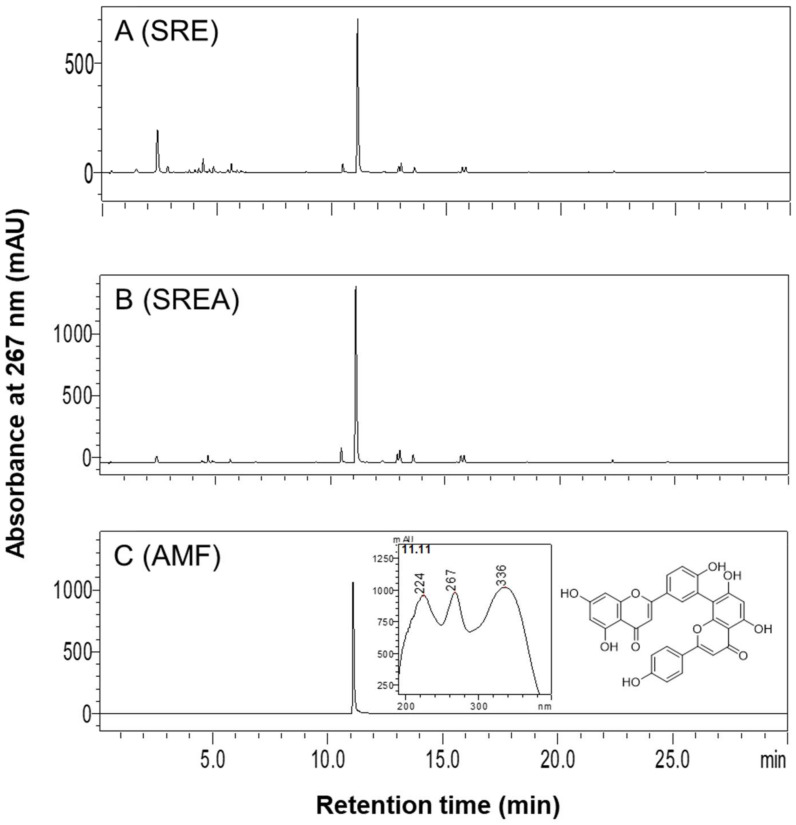
High-performance liquid chromatography (HPLC) profiles of 95% EtOH extract (SRE) and EtOAc extract (SREA) of SR and amentoflavone (AMF). Chromatograms of HPLC for (**A**) SRE (5 mg/mL), and (**B**) SREA (5 mg/mL) were detected at 267 nm. (**C**) Chromatogram of HPLC, UV spectrum, and chemical structure of AMF.

HPLC profiles of SRE, SREA, and AMF detected by HPLC-DAD at 267 nm are shown in Figure 1A–C. AMF (C_30_H_18_O_10_), a biflavonoid, was primarily present in SRE and SREA. The contents of AMF in SRE and SREA, quantified using the standard curve, were 66.6 mg/g and 154.8 mg/g, respectively. Compared with the extracts of other *Selaginellaceae* species, such as *S. tamariscina* and *S. involvens*, SR extracts showed much higher AMF contents (Figure S1 and Table S2).

### 3.2. Short-Term Administration of SR Suppressed Intestinal Lipid Absorption 

**Figure 2:**
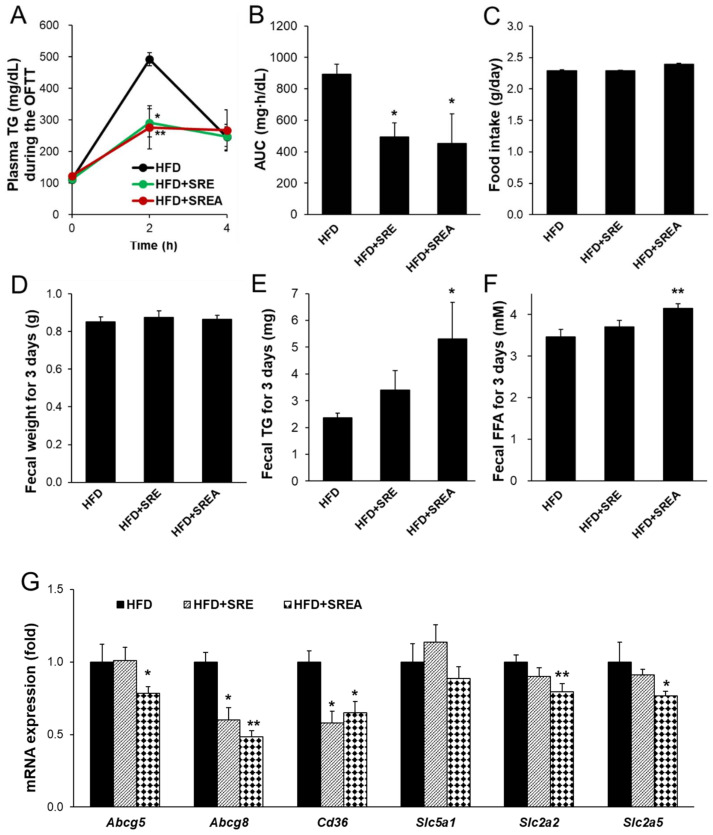
Effects of SR extracts on the intestinal lipid absorption in the HFD-fed mice. (**A**) After SR extracts were administrated for seven days, a fat absorption test was performed after orogastric gavage of olive oil. Plasma TG concentration was measured at 0–4 h. (**B**) Area under the curve (AUC) of plasma TG concentration during the oral fat absorption test. (**C**) The food intake was measured during oral gavage of SR extracts for seven days. Fecal weight (**D**), fecal triglyceride (TG) (**E**), and free fatty acid (FFA) (**F**) contents were measured using the total fecal collected over 3 days. (**G**) The intestinal mRNA expressions were measured by quantitative real-time RT-PCR (qRT-PCR). Values are given as means ± SE (*n* = 7). * *p* < 0.05, ** *p* < 0.01 vs. the HFD group.

After short-term (seven-day) administration of SR extracts, lipid absorption was detected using an oral fat tolerance test (OFTT). Administration of SR extracts significantly reduced the lipid absorption ability compared to that of the control (Figure 2A). The area under the curve (AUC) of the plasma TG concentration was significantly reduced in the HFD+SRE and HFD+SREA groups compared to that in the HFD group (Figure 2B). However, there were no differences in food intake or fecal weight among the three groups (Figure 2C,D). Fecal lipids were measured; the fecal TG and free FFA contents were significantly increased in the HFD+SREA group, although there was a trend toward increased fecal TG and FFA contents in the HFD+SRE group compared with that in the HFD group (Figure 2E,F). Additionally, lipid, carbohydrate, and protein absorption-related intestinal gene expression were measured. The lipid absorption- and transport-related gene expression of ATP-binding cassette subfamily G member 8 (*Abcg8*) and *Cd36* were significantly reduced in both SR extract-supplemented groups compared to that in the HFD group (Figure 2G). *Abcg5* expression was also significantly reduced in the HFD+SREA group compared to that in the HFD group. The carbohydrate absorption–related gene expression of solute carrier family 2 member 2 (*Slc2a2*) and *Slc2a5* were significantly reduced in the HFD+SREA group compared to the HFD group (Figure 2G). However, *Slc5a1* expression in the SR extract–fed groups did not change compared to that in the HFD group. The protein absorption–related gene expression of *Slc15a1*, *Slc8a2*, and *Slc6a9* was not altered in either SR extract–treated groups (Figure S2). These results indicate that short-term administration of SR extracts markedly suppressed intestinal lipid absorption by downregulating lipid absorption-related gene expression. 

### 3.3. Long-Term Administration of SR Reduced HFD-Induced Obesity

**Figure 3:**
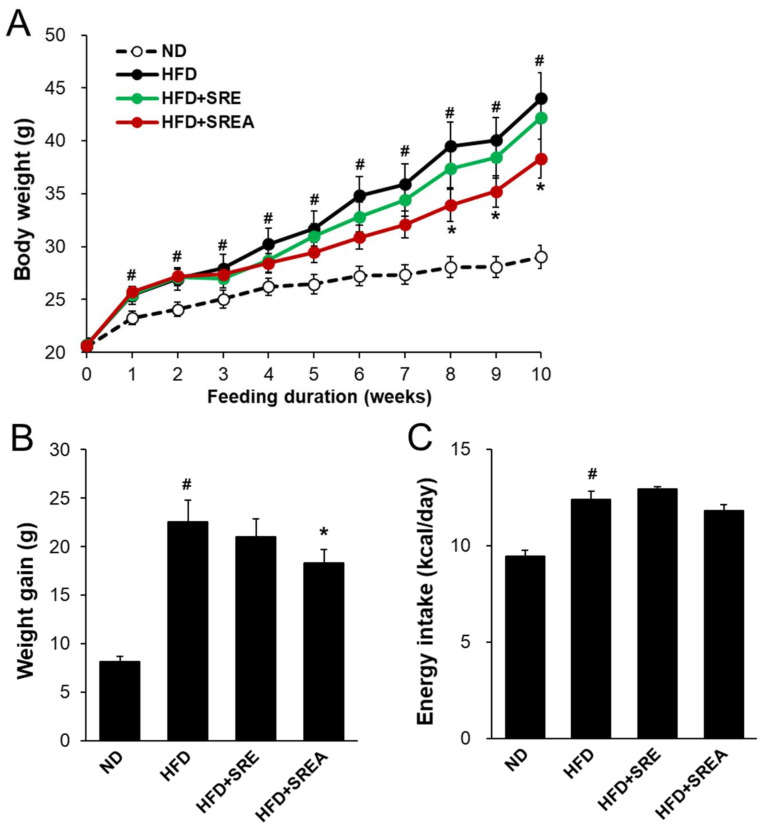

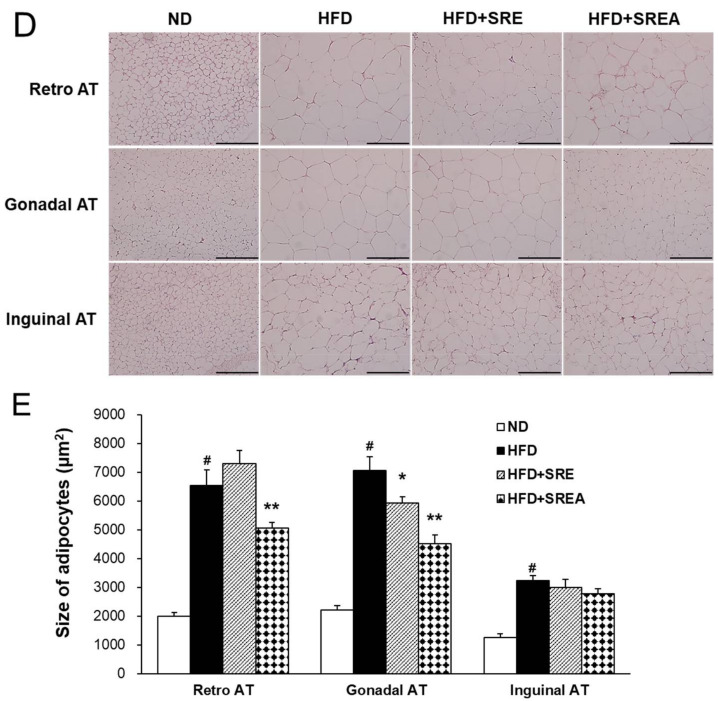
Effects of SR extracts on the body weight gain in the HFD-fed mice. (**A**) The body weights were monitored weekly during treatment of SR extracts for 10 weeks. (**B**,**C**) Weight gain and energy intake. (**D**) Images of the white adipose tissues (WATs) stained with H&E (×200 magnification, scale bar: 200 μm). (**E**) Adipocyte sizes of WATs. Values are given as means ± SE (*n* = 8). ^#^ *p* < 0.01 vs. the ND group; * *p* < 0.05, ** *p* < 0.01 vs. the HFD group. Retro AT, retroperitoneal AT.

Next, the anti-obesity effects of long-term SR extract administration were analyzed after 10-week feeding with SR extracts. The HFD group showed markedly increased body weight compared to that of the ND group (Figure 3A). The bodyweight of the HFD+SREA group decreased with statistical significance from eight weeks compared to that of the HFD. After 10 weeks, the bodyweight of the HFD+SREA group decreased by 19.0% compared with that of the HFD group (Figure 3B). However, energy intake did not differ between the groups receiving HFD (Figure 3C). At the end of the experiment, the organ weights of the liver, pancreas, and ATs were evidently higher in the HFD group than in the ND group (Table 1). Both SR extracts significantly decreased the retroperitoneal, inguinal, and total AT weights compared to that seen in the HFD group. In addition, the liver weights in the HFD+SREA group were significantly lower than those in the HFD group.

H&E staining revealed that the mean retroperitoneal and gonadal adipocyte sizes in the HFD+SREA group were significantly lower than in the HFD group (Figure 3D,E). In addition, the mean gonadal adipocyte sizes in the HFD+SRE group were markedly lower than in the HFD group. However, the mean inguinal adipocyte sizes did not change in SR extract-treated groups compared to the HFD group. Therefore, long-term supplementation with SR extracts can suppress HFD-induced adipocyte hypertrophy in visceral AT.

### 3.4. Long-Term Administration of SR Improved Metabolic Parameters in Plasma

**Figure 4:**
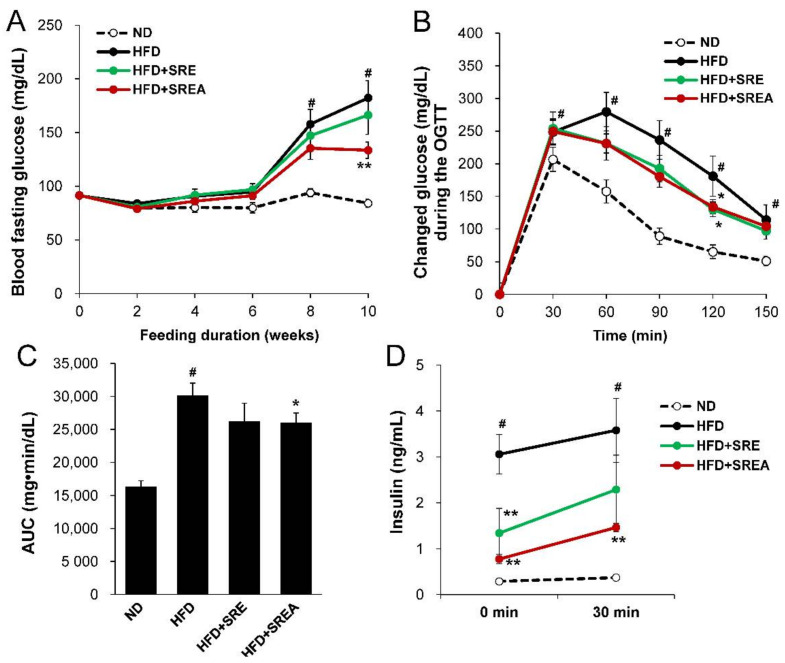

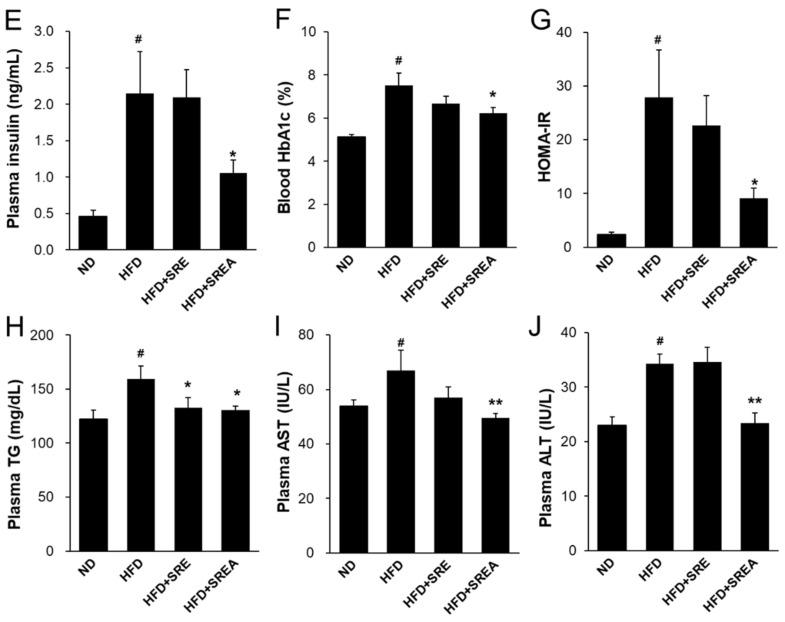
Effects of SR extracts on glucose homeostasis and lipid profiles. (**A**) Fasting glucose levels were monitored during SR extracts treatment. (**B**) OGTT was performed by oral gavage of glucose. The blood glucose concentration was measured at 0–150 min. The AUCs of glucose concentration (**C**) and plasma insulin concentration (**D**) were measured during OGTT. The plasma insulin (**E**), blood glycated hemoglobin (HbA1c) (**F**), homeostasis model assessment of insulin resistance (HOMA-IR) (**G**), plasma TG (**H**), plasma aspartate transaminase (AST) (**I**), and alanine transaminase (ALT) (**J**) were analyzed at the end of the experiment. Values are given as means ± SE (*n* = 8). ^#^ *p* < 0.01 vs. the ND group; * *p* < 0.05, ** *p* < 0.01 vs. the HFD group.

The fasting glucose concentration gradually increased after eight weeks in the HFD group compared with that in the ND group during long-term administration of SR extracts (Figure 4A). After 10 weeks, the glucose concentration in the HFD+SREA group was markedly lower (26.8%) than in the HFD group. In the OGTT, the glucose concentration in the HFD group was evidently higher at 30, 60, 90, 120, and 150 min compared with that in the ND group (Figure 4B). In addition, the glucose concentration in SR extract-treated groups was lower and statistically significant compared with those in the HFD group at 120 min. The impaired glucose tolerance in the HFD group also exhibited a higher AUC of glucose concentration compared to that in the ND group (Figure 4C). The AUC levels demonstrated a significant decline of 13.7% in the HFD+SREA group compared to that in the HFD group. The HFD group showed insulin resistance with higher plasma insulin concentration compared to that in the ND group at 0 and 30 min during OGTT (Figure 4D). Despite similar fasting glucose levels in all HFD-fed groups at 0 min during OGTT, the HFD+SRE and HFD+SREA groups showed markedly lower plasma insulin levels than the HFD group. Insulin concentration in the HFD+SREA group was lower (59.2%) than those in the HFD group at 30 min after glucose administration. 

After 10 weeks, the HFD group showed hyperglycemia and hyperlipidemia, which was confirmed by analyzing the plasma profiles (Figure 4E–H). However, the plasma insulin, blood glycated hemoglobin (HbA1c), and homeostasis model assessment of insulin resistance (HOMA-IR) levels were markedly lower in the HFD+SREA group by 51.2%, 17.3%, and 67.3%, respectively, compared to that in the HFD group. Plasma TG concentrations in the HFD+SRE and HFD+SREA groups significantly decreased by 16.6% and 17.9%, respectively, compared with that in the HFD group (Figure 4H). In addition, AST and ALT levels were significantly lowered by 26.0% and 31.8%, respectively, in the HFD+SREA group compared to that in the HFD group (Figure 4I,J). Overall, these results suggest that administering SR extracts protected against HFD-induced hyperglycemia and hyperlipidemia.

### 3.5. Long-Term Administration of SR Suppressed Intestinal Lipid Absorption

**Figure 5:**
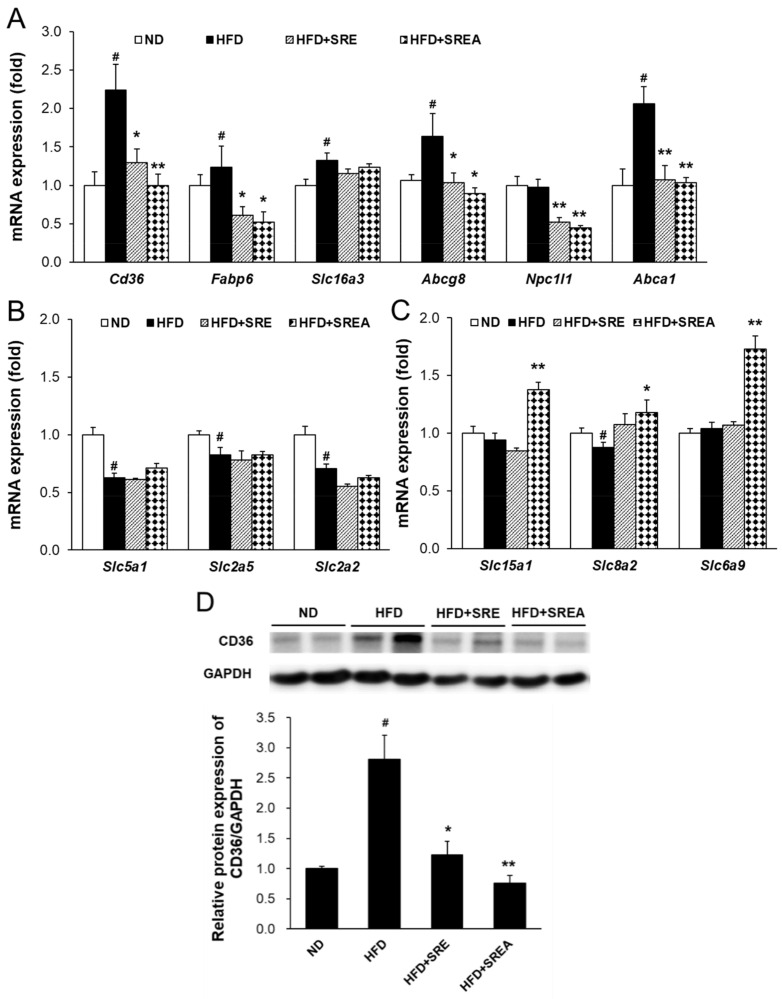
Effects of SR extracts on intestinal lipid absorption and intestinal protein absorption. The mRNA expression levels related to lipid (**A**), carbohydrate (**B**), and protein (**C**) absorption were measured by real-time qRT-PCR. (**D**) The CD36 protein expression levels were measured using anti-CD36 and anti-GAPDH antibodies. Values are given as means ± SE (*n* = 8). ^#^ *p* < 0.01 vs. the ND group; * *p* < 0.05, ** *p* < 0.01 vs. the HFD group.

Next, intestinal mRNA expressions were measured to assess nutritional absorption levels. Expressions of lipid absorption–related genes, including *Cd36*, fatty acid-binding protein 6 (*Fabp6*), *Abcg8*, Niemann-Pick C1 like 1 (*Npc1l1*), and ATP-binding cassette transporter A1 (*Abca1*), in the SR extract-treated groups were decreased with statistical significance than those in the HFD group (Figure 5A). The expressions of carbohydrate absorption-related genes, including *Slc5a1*, *Slc2a2*, and *Slc5a2*, in the SR extract-fed groups, did not change compared to that in the HFD group (Figure 5B). In addition, the expression of protein absorption-related genes, including *Slc15a1*, *Slc8a2*, and *Slc6a9*, in the HFD+SREA group were markedly increased compared with that in the HFD group (Figure 5C). CD36 protein expressions in the SR extract-fed groups were also markedly decreased when compared to that in the HFD group (Figure 5D). Therefore, these findings indicate that SR extracts suppress intestinal lipid absorption and enhance protein absorption in mice.

### 3.6. Long-Term Administration of SR Protected Development of Hepatic Steatosis

**Figure 6:**
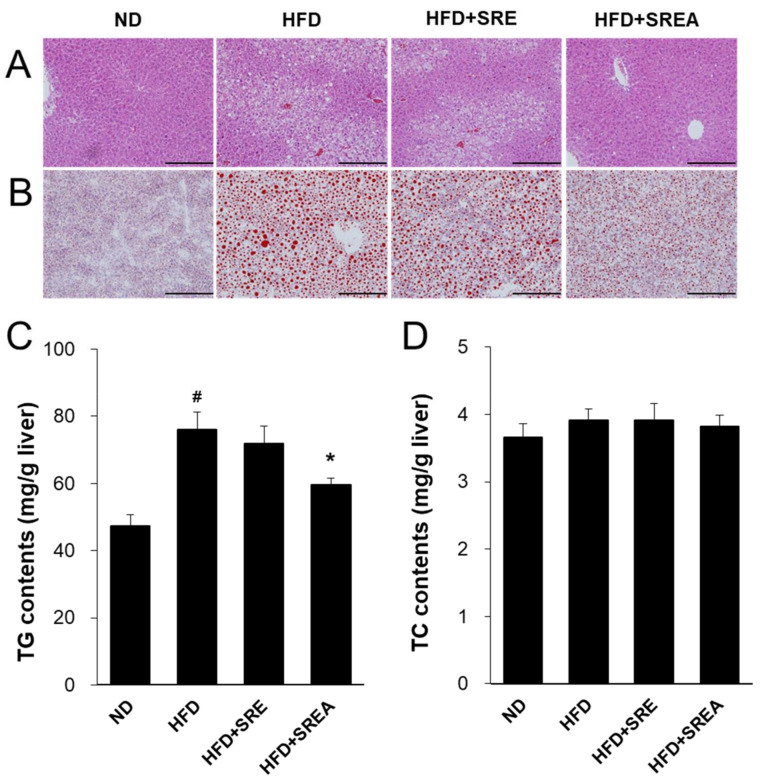

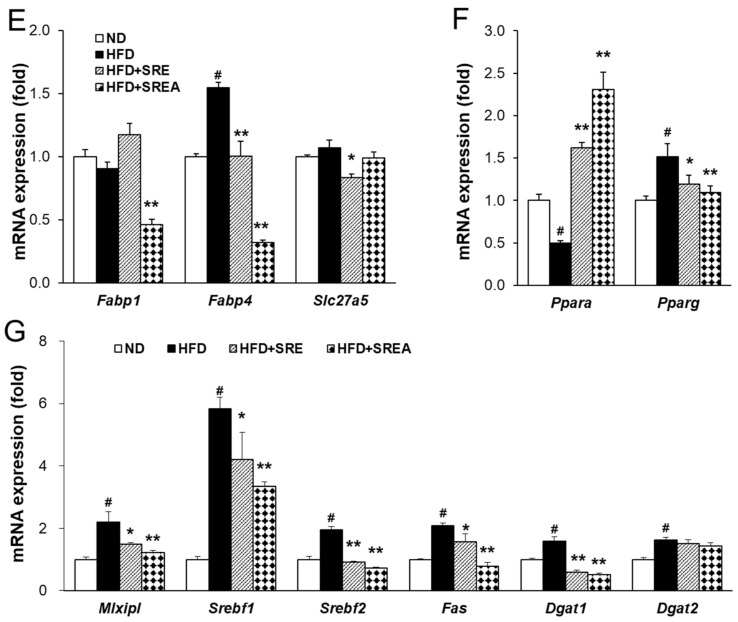
Effects of SR extracts on hepatic steatosis. (**A**,**B**) Images of liver were stained by H&E and Oil-Red O (× 200 magnification, scale bar: 200 μm). Hepatic TG (**C**) and TC (**D**) contents. (**E**–**G**) The hepatic mRNA expression was measured by qRT-PCR. Values are given as means ± SE (*n* = 8). ^#^ *p* < 0.01 vs. the ND group; * *p* < 0.05, ** *p* < 0.01 vs. the HFD group.

Suppressed lipid absorption can protect against obesity development and lipid deposition in organs; thus, hepatic steatosis was assessed after long-term administration of the SR extracts. Histological analysis revealed that the number of ballooned hepatocytes and lipid droplets was markedly increased in the HFD group compared with that in the ND group (Figure 6A,B). In the SR extract-treated groups, hepatocellular ballooning and lipid droplets were markedly decreased compared to the HFD group. Hepatic TG content was lower and statistically significant in the HFD+SREA group than in the HFD group (Figure 6C). However, there were no differences in hepatic TC content among all groups (Figure 6D). Treatment with SREA significantly reduced the mRNA expression of fatty acid transport-related genes, including *Fabp1* and *Fabp4*, compared with that in the HFD group (Figure 6E). Administration of SRE extract significantly decreased the mRNA levels of fatty acid transport-related *Fabp4* and *Slc27a5* compared to that in the HFD group. Long-term supplementation of both SR extracts significantly increased the mRNA levels of *Ppara* and decreased *Pparg* compared to those in the HFD group (Figure 6F). In the HFD+SRE and HFD+SREA groups, the mRNA levels of hepatic de novo lipogenesis–related transcription factors and their target genes were significantly decreased, including MLX interacting protein-like (*Mlxipl*), sterol regulatory element-binding transcription protein 1 (*Srebf1*), *Srebf2*, fatty acid synthase (*Fas*), and diacylglycerol O-acyltransferase 1 (*Dgat1*) (Figure 6G). Hence, SR extracts can protect against HFD-induced hepatic steatosis by inhibiting lipid transport and biosynthesis. 

### 3.7. AMF Suppressed Intestinal Lipid Absorption 

**Figure 7:**
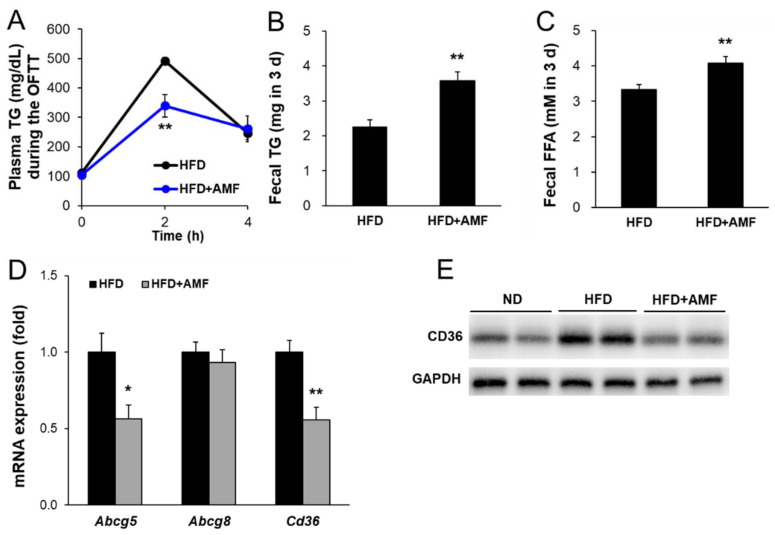
Effects of amentoflavone (AMF) on intestinal lipid absorption. (**A**) After AMF were administrated for seven days, a fat absorption test was performed after overnight fasting. Mice were given an orogastric gavage of olive oil. The plasma TG concentration was measured at 0, 2, and 4 h. The fecal TG (**B**) and FFA (**C**) contents were measured using the total fecal collected over 3 days. (**D**) The intestinal mRNA levels were measured by qRT-PCR. (**E**) The intestinal CD36 protein levels were measured using anti-CD36 and anti-GAPDH antibodies. Values are given as means ± SE (*n* = 6). * *p* < 0.05, ** *p* < 0.01 vs. the HFD group.

To determine whether enriched AMF could suppress intestinal lipid absorption, the effects of AMF on OFTT, fecal lipid, and intestinal mRNA and protein expression in HFD-fed mice were examined. Oral treatment with AMF (HFD with 10 mg∙kg^−1^∙day^−1^ AMF) for seven days resulted in significantly lower plasma TG levels in the HFD+AMF group than in the HFD group at 2 h after oil gavage (Figure 7A). The fecal TG and FFA contents were significantly higher in the HFD+AMF group than in the HFD group (Figure 7B,C). The mRNA levels of lipid absorption-related genes, including *Abcg5* and *Cd36*, were significantly decreased in the HFD+AMF group compared to the HFD group but did not change in *Abcg8* expression (Figure 7D). In addition, CD36 protein expression levels were markedly lower in the HFD+AMF group than in the HFD group (Figure 7E). These results indicate that AMF may contribute to suppressing intestinal lipid absorption by SR extracts.

## 4. Discussion

In this study, we determined that SR extracts protected against HFD-induced obesity, hyperglycemia, and hepatic lipid accumulation in mice. AMF may function as an active compound in SR extracts. Previous studies have determined that AMF inhibits α-glucosidase and DPP-4 on in vitro enzyme activities [23,24] but does not inhibit pancreatic lipase activity [25]. AMF reduces HFD-induced obesity and hyperglycemia by regulating lipogenesis and insulin signaling [19,20] and streptozotocin-induced hyperglycemia by activating the PI3K/Akt signaling pathway in mice [26]. AMF has been shown to improve cardiovascular dysfunction with lipid ectopic distribution in mice fed an HFD [27]. The AMF contents of the methanol (MeOH) extracts in nine species of *Selaginellaceae* were determined by reversed-phase HPLC. Among them, the MeOH extract of *S. tamariscina* and *S. sinensis* showed the AMF contents at 0.70% and 1.13%, respectively [28]. Our results confirmed that SR contained much higher contents of AMF compared to those recorded in other *Selaginellaceae* species such as *S. tamariscina* and *S. involvens* (Figure S1 and Table S2). The glucose- and lipid-lowering activity of the SREA group (7.74 mg∙kg^−1^∙day^−1^ AMF in 50 mg∙kg^−1^∙day^−1^ SREA) was significantly superior to that of the SRE group (6.66 mg∙kg^−1^∙day^−1^ AMF in 100 mg∙kg^−1^∙day^−1^ SRE) in HFD-fed mice. Therefore, a high dose of AMF may have contributed to the anti-obesity and glucose-lowering activity of SR extracts in HFD-fed mice. To the best of our knowledge, this is the first study reporting that the glucose- and lipid-lowering activities of AMF may be caused by the suppression of intestinal lipid absorption in HFD-fed mice. 

Digested dietary nutrients, including carbohydrates, fats, and proteins, are absorbed from the intestinal lumen into the blood circulation. Dietary lipids, including TGs, phospholipids, and cholesterol esters, are absorbed and secreted from the intestine, which is important for maintaining whole-body energy homeostasis and possess major impacts on health and diseases [7]. During lipid absorption, CD36 and FATPs regulate fatty acid absorption, and NPC1L1 primarily regulates cholesterol transport to intestinal enterocytes [29,30]. CD36 deficiency leads to lipid accumulation in the small intestine by decreasing fatty acid transport to the lymphatic system [31]. Transported fatty acids in enterocytes bind to FABPs to migrate to the endoplasmic reticulum for re-synthesis of TG and further output of pre-chylomicrons [32,33]. Conversely, ABCG5 and ABCG8 transporters efflux cholesterol from enterocytes into the intestinal lumen [34]. ABCA1 mediates cytosolic cholesterol transport to blood circulation for high-density lipoprotein biosynthesis from dietary cholesterol absorption by interacting with caveolin 1 and scavenger receptor class B type I [35,36]. In this study, we observed that both short- and long-term administration of SR extracts suppressed intestinal fatty acid and monoacylglycerol absorption by inhibiting *Cd36* expression (Figure 2G and Figure 5A,D). Long-term administration of SR extracts also inhibited cholesterol uptake and fatty acid transport by suppressing *Npc1l1* and *Fabp6* expression (Figure 5A). Additionally, the SR extracts suppressed cholesterol efflux from enterocytes by inhibiting the expression of *Abcg8* and *Abca1* (Figure 5A). This may be caused by the decreased lipid source via the suppression of lipid absorption by enterocytes. AMF reduces CD36 expression with inhibition of PPARγ in oxidized low-density lipoprotein-induced human umbilical artery smooth muscle cells [37]. In this study, AMF acted as an active component and significantly reduced intestinal CD36 expression with suppression of lipid absorption and enhancement of lipid excretion (Figure 7).

Dietary digested carbohydrates are primarily absorbed as monosaccharides, including glucose, galactose, and fructose. Glucose and galactose are transported to enterocytes via sodium-glucose cotransport 1 down a sodium osmotic gradient [38]. Fructose is absorbed by enterocytes through GLUT2 (encoded by *Slc2a2*) and GLUT5 (encoded by *Slc2a5*) [39]. Dietary proteins are digested into amino acids and small peptides through various gastrointestinal proteases, including pepsin, trypsin, chymotrypsin, and elastase. Digested small peptides, including dipeptides and tripeptides, are efficiently absorbed via peptide transporter 1 (PEPT1, encoded by *Slc15a1*). In a diabetic environment, intestinal PEPT1 expression and its function are downregulated by chenodeoxycholic acid-mediated farnesoid X receptor activation [40]. The impaired PEPT1 function can be reversed by leptin supplementation in *ob*/*ob* mice [41]. All neutral amino and amino acids are transported to enterocytes by sodium-coupled neutral amino acid transporter 2 (encoded by *Slc8a2*). Intestinal transport of glycine was primarily conducted by glycine transporter 1 (GLYT1, encoded by *Slc6a9*) for glutathione and creatinine synthesis. GLYT1 exerts a potential role in the protection against intestinal oxidative stress and inflammatory diseases [42]. In this current study, short-term administration of SR extracts may decrease monosaccharide absorption by inhibiting *Slc2a2* and *Slc2a5* (Figure 2G). However, long-term administration of SR extracts did not change the expression of monosaccharide absorption–related genes, including *Slc5a1*, *Slc2a2*, and *Slc2a5* (Figure 5B). Further, short-term administration of SR extracts did not change the intestinal absorption of protein source–related gene expression (Figure S2); however, long-term supplementation with SREA significantly increased protein absorption-related gene expression, including *Slc15a1*, *Slc8a2*, and *Slc6a9* (Figure 5C). These results suggest that chronic suppression of lipid absorption may promote carbohydrate or protein absorption for energy homeostasis. 

Chronic and excessive lipid absorption induces hyperlipidemia, leading to lipotoxicity in peripheral lipid storage tissues such as ATs, liver, and muscles [43,44] promotes hepatic lipid accumulation accompanied with up-regulation of lipogenic gene expression including *Srebf1*, *Pgc1b*, *Acc1*, *Acc2*, *Fas*, *Dgat1*, *Dgat2*, and *Gpat* [45]. In this study, the HFD group showed fatty liver phenotypes with increased TG contents and expression of upregulated de novo lipogenesis-related genes. However, SR treatment attenuated HFD-induced hepatic steatosis by suppressing TG contents and the expression of fatty acid transport- and lipogenesis-related genes including *Fabp1*, *Fabp4*, *Slc27a5*, *Pparg*, *Mlxipl*, *Srebf1*, *Srebf2*, *Fas*, *Dgat1*, and *Dgat2* (Figure 6). These results suggest that SR treatment prevented hepatic steatosis by reducing lipid sources through long-term suppression of intestinal lipid absorption. In addition, long-term (8–17 weeks) supplementation of AMF suppresses hepatic steatosis with decreasing lipogenesis-related gene expression, including *Cebpa*, *Fabp4*, and *Slc27a4* in HFD-fed mice [19,26,27]. Feeding HFD develops obese conditions by storing excess energy in both visceral and subcutaneous ATs. Visceral ATs show higher lipolysis and lower lipogenesis compared to that of subcutaneous ATs [46]. Visceral obesity contributes to the development of hepatic steatosis and hepatic insulin resistance by direct exposure to increased FFA content [47]. Supplementation with SR extracts significantly reduced adipocyte hypertrophy in visceral ATs, including retroperitoneal and gonadal ATs (Figure 3). These results suggest that SR treatment attenuated HFD-induced adipocyte hypertrophy in visceral ATs; it may contribute to improving hepatic steatosis, de novo lipogenesis, and glucose homeostasis (Figure 4). 

Our findings demonstrated that SR extracts regulated the intestinal absorption of lipids, carbohydrates, and proteins. Especially, SR extracts suppressed lipid absorption in short-term and long-term supplementation. AMF may be an active compound of SR extracts based on its ability to inhibit lipid absorption. Furthermore, SR extracts reduced ectopic lipid accumulation in the liver and ATs by reducing the lipid resources in vivo via inhibiting intestinal lipid absorption. These regulatory functions of SR extracts may be caused by alterations in the gut microbiota composition. AMF inhibits gut bacterial β-glucuronidase activity and can treat drug-induced enteropathy [48]. Thus, AMF-enriched SR extracts may alter the gut microbiota composition to regulate lipid absorption in the gut. Further studies are needed to clarify whether gut microbiota composition and metabolites regulated by SR extracts modulate gut function. On the other hand, there is no information against other compounds of SR compared to that for other *Selaginellaceae* species [17,49]. Hence, further studies need to investigate other active compounds of SR for its further application.

In summary, this study evaluated the effects of SR extracts in HFD-fed obese and diabetic mice. Short-term administration of SR extracts effectively reduced intestinal lipid absorption and increased lipid excretion in the feces of HFD-fed mice. Long-term supplementation with SR extracts significantly reduced HFD-induced body weight gain by suppressing adipocyte hypertrophy in visceral AT. Furthermore, long-term supplementation with SR extracts effectively protected against HFD-induced hyperlipidemia and improved glucose homeostasis. SR extract supplementation also protected against HFD-induced hepatic steatosis by inhibiting the expression of fatty acid transporters and de novo lipid lipogenesis in the liver. Overall, the effects of SR extracts were primarily caused by the suppression of intestinal lipid absorption via inhibition of CD36, *Fabp6*, *Npc1l1*, and *Abca1* expression. Importantly, AMF was identified as an active component of SR and markedly suppressed intestinal lipid absorption via inhibition of CD36 expression with *Abcg5*. This study investigated the lipid- and glucose-lowering effects of SR and whether these effects may be caused by suppression of intestinal lipid absorption. Taken together, the findings indicate that SR could be used as a novel and good treatment option for diet-induced obesity and diabetes.

## Figures and Tables

**Table 1:** Effects of SR extracts on organ weights in HFD-fed mice.

Organs (g/kg body Wt.)	ND	HFD	HFD+SRE	HFD+SREA
Liver	26.0 ± 1.2	30.1 ± 2.1 ^#^	27.4 ± 0.8	25.5 ± 0.7 *
Pancreas	4.1 ± 0.2	4.5 ± 0.3	4.1 ± 0.3	4.0 ± 0.2
WATs				
Retroperitoneal AT	5.8 ± 1.0	28.1 ± 2.7 ^#^	22.6 ± 1.4 *	22.4 ± 1.6 *
Gonadal AT	20.8 ± 2.2	56.3 ± 2.9 ^#^	56.5 ± 3.1	55.3 ± 4.6
Inguinal AT	14.7 ± 1.8	46.3 ± 2.1 ^#^	36.1 ± 3.6 *	34.5 ± 2.4 **
Total WAT	41.3 ± 4.8	122.7 ± 3.1 ^#^	112.6 ± 6.5 *	110.9 ± 7.6 *

Values are given as means ± SE, *n* = 8. ^#^ *p* < 0.01 vs. the ND group; * *p* < 0.05, ** *p* < 0.01 vs. the HFD group. WAT, white adipose tissue; AT, adipose tissue.

## Data Availability

Data are included in the article or Supplementary Material.

## Supplementary Materials

The following supporting information can be downloaded at: https://www.mdpi.com/article/10.3390/life12040472/s1, Figure S1: HPLC profiles of extracts of *S.* *rossii* and other *Selaginella**ceae* species. Figure S2: Short-term supplementation of SR extracts did not change intestinal protein absorption-related gene expressions. Table S1: Primers used for mRNA quantification. Table S2: AMF contents of *S. rossii* and other *Selaginellaceae* species.
