# Supplementary material for: Amentoflavone-Enriched Selaginella rossii Warb. Suppresses Body Weight and Hyperglycemia by Inhibiting Intestinal Lipid Absorption in Mice Fed a High-Fat Diet
Source: Life (Basel). 2022 Mar 24;12(4):472. doi: 10.3390/life12040472 (PMC9024644; doi:10.3390/life12040472)
Supplement: Supplementary file 1 [file life-12-00472-s001.zip › life-1604142-supplementary.pdf]

Supplementary file

Amentoflavone-Enriched *Selaginella rossii* Warb. Suppresses Body Weight and Hyperglycemia by Inhibiting Intestinal Lipid Absorption in Mice Fed a High-Fat Diet

Hwa Lee [1,†], Seona Cho [1], Soo-Yong Kim [2], Jeongha Ju [1,3], Sang Woo Lee [2], Sangho Choi [2], Hulin Li [4], Renzhe Piao [4], Ho-Yong Park [1] and Tae-Sook Jeong [1,*]

[1] Industrial Bio-Materials Research Center, Korea Research Institute of Bioscience and Biotechnology (KRIBB), Daejeon 34141, Korea; leehua@kribb.re.kr (H.L.); baby5624@naver.com (S.C.); wjdgk0605@naver.com (J.J.); hypark@kribb.re.kr (H.-Y.P.)
[2] International Biological Material Center, KRIBB, Daejeon 34141, Korea; soodole@kribb.re.kr (S.-Y.K.); ethnolee@kribb.re.kr (S.W.L.); decoy0@kribb.re.kr (S.C.)
[3] Department of Medical Biotechnology, Yeungnam University, Gyeongsan 38541, Korea
[4] Department of Agronomy, Agriculture College of Yanbian University, Yanji 133000, China; lhlsym@ybu.edu.cn (H.L.); rzpiao@ybu.edu.cn (R.P.)
* Correspondence: tsjeong@kribb.re.kr; Tel.: +82-42-860-4558
† Earlier known as Hua Li.

1. Primer sequences used in this article are described in Table S1.

Table S1. Primers used for mRNA quantification.

Gene Name	Forward Primer	Reverse Primer
Abca1 (NM_013454)	TCAATGGAAGGTTCAGGTGC	TCATCACTTTGGTCCTTGGC
Abcg5 (NM_031884)	TCTGTTTCCCATGCTGAGAG	CGGGTGATTTAGCATAGAGG
Abcg8 (NM_026180)	CAATGTCATCCTGGATGTCG	ATGGAGAAGGTGAAGTTGCC
Cd36 (NM_007643)	TGCTGGAGCTGTTATTGGTG	CTCAAAGATGGCTCCATTGG
Dgat1 (NM_010046)	ACAACCTGACCTACCGAGAT	AGTAGGGACCATCCACTGTT
Dgat2 (NM_026384)	GCTGGCATTTGACTGGAACA	TGGTCAGCAGGTTGTGTGTCTT
Fabp1 (NM_017399)	GCAAGTACCAATTGCAGAGCCAGG	TCATTGCGGACCACTTTGGG
Fabp4 (NM_024406)	TTTGTGGGAACCTGGAAGCT	CACGCCCAGTTTGAAGGAAA
Fabp6 (NM_008375)	TCTTCCAGGAGACGTGATTG	TACGCGCTCATAGGTCACAT
Fas (NM_007988)	TGTGAGTGGTTCAGAGGCAT	TTCTGTAGTGCCAGCAAGCT
Gapdh (NM_001001303)	ACATCATCCCTGCATCCACT	AGATCCACGACGGACACATT
Hmgcr (NM_008255)	TAGGCTTGGTCCTTGTTCAC	GCTTTCTTTGAGGTCACGAC
Mlxipl (NM_021455)	CAGATGCGGGACATGTTTGA	AATAAAGGTCGGATGAGGATGCT
Npc1l1 (NM_207242)	ACGGAACTCACAGGACTTTACAGAA	TTGCTGGTAGAACACATTGGAGAT
Nr1h3 (NM_013839)	TGCCCCATGGACACCTACA	CTTGCCGCTTCAGTTTCTTCA
Pparα (NM_011144)	CCTGAACATCGAGTGTCGAA	GTACTGGCATTTGTTCCGGT
Pparg (NM_001127330)	ATGTCTCACAATGCCATCAGGTT	GCGGGAAGGACTTTATGTATGAGT
Slc2a2 (NM_031197)	TTTGTCATCGCCCTCTGCTT	GCAGCGATTTCCTCAAAAGACT
Slc2a5 (NM_019741)	GTGTCCACTGGCTCTCTAACTTCA	GGTGGTGAGGAAACAGATGGTT
Slc5a1 (NM_019810)	GCCTCTCTCTTTGCCAGTAACATT	CAAGGCGTTCCATTCAAAGC
Slc6a9 (NM_001355175)	CACTGCCATTGTGGATGAGGTA	GATACAGGAGATGACAACCAAGGA
Slc8a2 (NM_148946)	TAGTGGATGATGAAGAGTATGAGAAGAAG	TCTTGTCTCCATTCCCTTGGTT
Slc15a1 (NM_053079)	GGTTACCCGTTGAGCATCTTCTT	TGCCATAGTAGGAGAATCTTTCACA
Slc16a3(NM_001038653)	GCGGTCAGCGTCTTTTTCAA	CCGTGTCGCTGTAGCCAAT
Srebf1 (NM_011480)	GAGCGAGCGTTGAACTGTAT	ATGCTGGAGCTGACAGAGAA
Srebf2 (NM_033218)	TCCTCCATCAACGACAAAATCA	ACTTGTGCATCTTGGCATCTGT

2. Comparison of amentoflavone (AMF) contents in extracts of *S. rossii* and other *Selaginellaceae* species

Preparation of SR extracts and HPLC Analysis

The dried aerial part of *Selagenella rossii* (Barker) Warb (SR) were obtained from Yanbian University (Yanji, China) delivered by the International Biological Material Research Center of the Korea Research Institute of Bioscience and Biotechnology (KRIBB, Daejeon, Korea). The 1 g dried SR powder was extracted using 10 mL MeOH at 20–25 ˚C for 48 h. The extract of MeOH was concentrated *in vacuo* to yield brown residue. The MeOH extracts of *Selaginella tamariscina* and *Selaginella involvens* were obtained from the Korea Plant Extract Bank (KPEB, Ochang, Korea).

The components of MeOH extracts of *Selaginellaceae* were analyzed using a high-performance liquid chromatography-diode array detector (HPLC-DAD) system (Shimadzu Corp., Tokyo, Japan). The extracts were separated on a Brownlee SPP C18 column (4.6 × 50 mm, 2.7 μm; Perkin Elmer, Inc, Waltham, MA, USA). The compositions of mobile phase are 0.1% acetic acid in water (mobile phase A) and acetonitrile (mobile phase B). The linear gradient elution program was as

follows: 5–50% B at 0–15 min, 50–100% B at 15–20 min, 100% B at 20–25 min, 100–5% B at 25–27 min, 5% B at 27–30 min. The absorbance of the HPLC profile was 267 nm, and the flow rate was 1.8 mL/min. Amentoflavone (AMF, Biopurify Phytochemicals Ltd., Chengdu, China) was used for analysis of AMF content.

Compared with other *Selaginellaceae* species such as *S. tamariscina* and *S. involvens*, SR extracts showed much higher AMF contents (Figure S1 and Table S2).

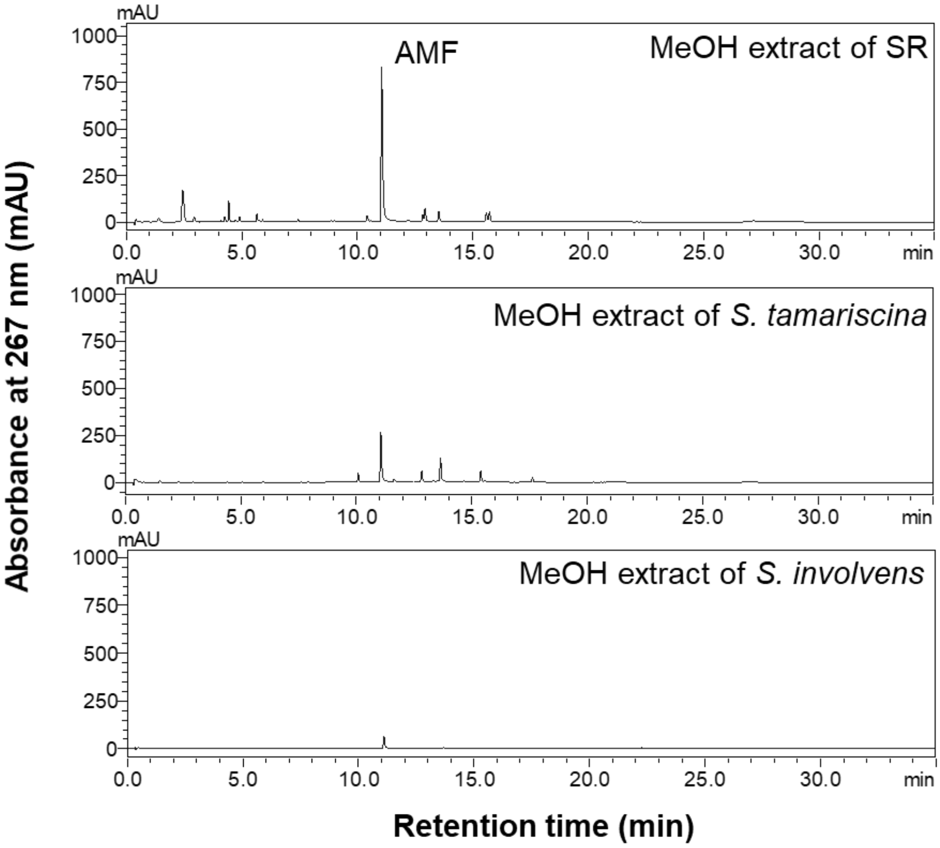

Figure S1. HPLC profiles of extracts of *S. rossii* (SR) and other *Selaginellaceae* species. Chromatograms of HPLC for methanol (MeOH) extract of SR, *S. tamariscina*, and *S. involvens* extracts (5 mg/mL) were detected at 267 nm.

Table S2. AMF contents of *S. rossii* and other *Selaginellaceae* species.

Samples	AMF Contents (mg/g extract)
EtOH extract of *S. rossii* (SRE)	66.6
EtOAc extract of *S. rossii* (SREA)	154.8
MeOH extract of *S. rossii*	89.2
MeOH extract of *S. tarmariscina*	25.8
MeOH extract of *S. involvens*	6.5

3. The protein absorption-related gene expressions of solute carrier family, including *Slc15a1*, *Slc8a2*, and *Slc6a9* were not changed in both SR extracts administered groups (Figure S2).

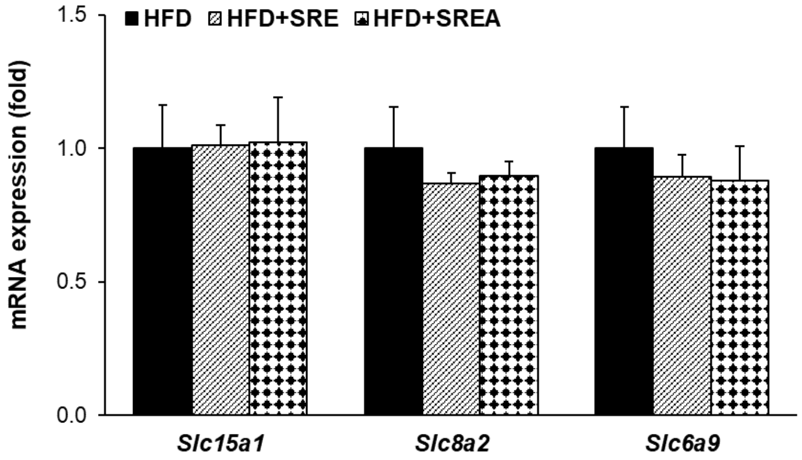

Figure S2. Short-term supplementation of SR extracts were not changed intestinal protein absorption-related gene expressions. The intestinal mRNA expression levels were measured by real-time qRT-PCR. Values are presented as means ± SE (*n* = 7).
